# Knotted and Encrusted Double J Stent: A Rare Complication Managed With Advanced Endoscopic Techniques

**DOI:** 10.1155/carm/8955098

**Published:** 2025-03-10

**Authors:** Seyed Reza Hosseini, Fardin Asgari, Mina Rezayat, Abdolreza Mohammadi, Seyed Mohammad Kazem Aghamir

**Affiliations:** Urology Research Center, Tehran University of Medical Sciences, Tehran, Iran

**Keywords:** case report, encrustation, endourology, knotted ureteral stent, minimally invasive surgery

## Abstract

Complications related to double J (DJ) stent placement are well documented, but DJ stent knotting remains an exceedingly rare event. We present a unique case of a knotted and encrusted DJ stent, alongside a review of the literature on this rare complication. A 55-year-old man with a history of ureteral obstruction due to stones was managed initially with percutaneous nephrostomy (PCN) and DJ stent placement. The patient presented after a delayed follow-up for stent removal. Despite imaging showing no evidence of encrustation or knotting, cystoscopic attempts to remove the stent failed. Further evaluation, including ureteroscopy, revealed significant encrustation and knotting of the DJ stent at the renal pelvis. The stent was successfully removed using a semirigid ureteroscope and lithoclast without open surgery. This case highlights the importance of early follow-up and patient compliance in preventing such complications. When simple traction fails to remove a DJ stent, advanced endoscopic techniques such as lithotripsy and ureteroscopy should be employed to manage complex cases effectively.

## 1. Introduction

Double J (DJ) stents are indispensable tools in the management of various urological conditions, facilitating urine drainage and preventing obstruction. Despite their utility, long-term stent placement is associated with complications such as encrustation, knotting, and fragmentation, which can lead to significant morbidity if not addressed promptly. Encrustation occurs in up to 76.3% of the cases with prolonged stent retention, influenced by urinary tract infections, metabolic abnormalities, and biofilm formation [1–5]. Knotting, though rare, poses a complex retrieval challenge requiring advanced endoscopic techniques or surgical interventions. Proper timing for stent removal and proactive follow-ups are critical in minimizing these complications [1, 6, 7].

## 2. Case Presentation

**Figure 1:**
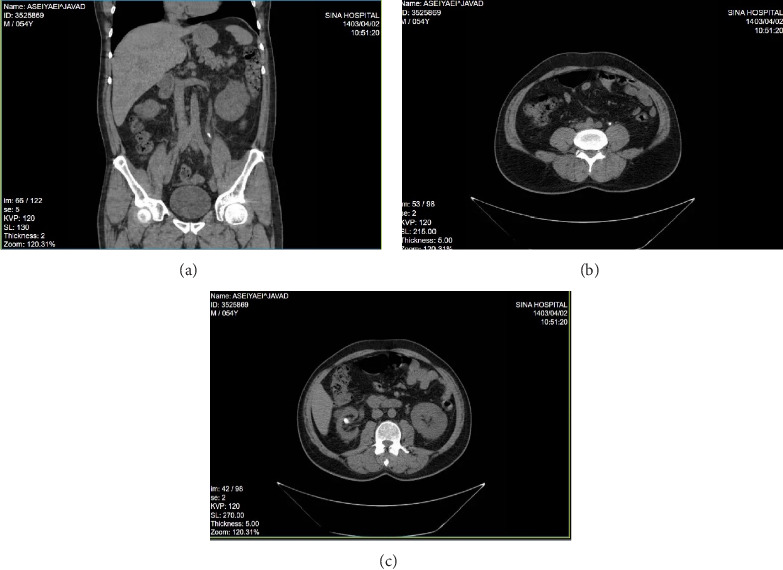
(a–c) Coronal and axial cut of noncontrast CT that revealed a 15 × 8 mm stone in the midureter of the left kidney, causing severe hydronephrosis (a-b). Axial cut of noncontrast CT that revealed a 13 × 10 mm stone in the left renal pelvis, causing mild hydronephrosis (c).

A 55-year-old male patient, weighing 85 kg and with a height of 178 cm, presented with severe left flank pain persisting for 21 days, associated with irritative urinary symptoms and gross hematuria. The patient denied any history of fever or chills and reported no prior medical or familial history of urological or systemic conditions. He worked as a laborer, with no history of smoking or alcohol consumption, and reported normal daily fluid intake. Upon physical examination, vital signs were stable, but costovertebral angle (CVA) tenderness was noted on the left side. Other physical findings were unremarkable. “A noncontrast CT scan was performed as the primary imaging modality, which was selected over ultrasonography due to its superior accuracy in detecting stone burden, hydronephrosis, and potential complications. No ultrasound was performed in this case.” revealing:

• A 15 × 8 mm stone in the midureter of the left kidney, causing severe hydronephrosis.
• A 13 × 10 mm stone in the right renal pelvis, causing mild hydronephrosis. (Figure 1)

Signs of inflammation in the left collecting system were also noted. Laboratory results showed elevated serum creatinine levels (6.39 mg/dL), confirming obstructive uropathy. Percutaneous nephrostomy (PCN) was promptly performed to relieve pressure in the left collecting system and improve renal function, as it was deemed more appropriate than DJ stent placement due to the presence of complete obstruction, significant hydronephrosis, and the need for immediate decompression to prevent further renal deterioration. The patient had PCN in place for 2 weeks before definitive treatment was performed. Following this intervention, serum creatinine levels normalized.

**Figure 2:**
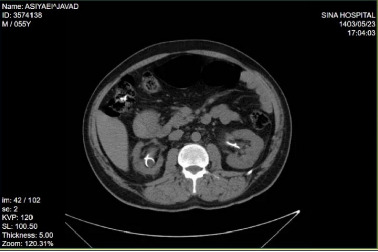
Noncontrast CT of abdominal pelvic with bilateral double J after surgery.

After 2 weeks, the patient underwent transurethral lithotripsy (TUL). However, due to significant ureteral stricture, a DJ stent was inserted on the left side to maintain patency. Given the mild hydronephrosis on the right side, another DJ stent was placed to assist with stone passage and prevent further obstruction, and extracorporeal shock wave lithotripsy (ESWL) was subsequently performed on the same side (for pelvic stone on right side) to facilitate stone fragmentation (Figure 2).

**Figure 3:**
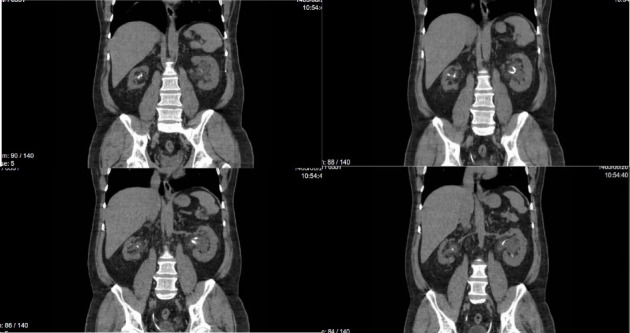
Noncontrast CT scan of abdominal pelvic showed a patent urinary tract with no evidence of encrustation or knotting in left kidney.

Four weeks poststent insertion, the patient returned with stable renal function for follow-up. The right DJ stent was removed and ureteroscopy was done and one piece of small stone extraction with basket successfully (probably that small fragment passage spontaneously after DJ insertion and ESWL), and the left ureteral stone was completely fragmented and cleared using lithoclast. However, significant inflammation and bolus were observed, necessitating the replacement of a new DJ stent on both side to ensure proper drainage and healing. The patient was discharged in stable condition with instructions to return for DJ stent removal in 4 weeks. Despite recommendations, the patient presented 2 months later for stent removal. A control CT scan showed a patent urinary tract with no evidence of encrustation or knotting (Figure 3).

**Figure 4:**
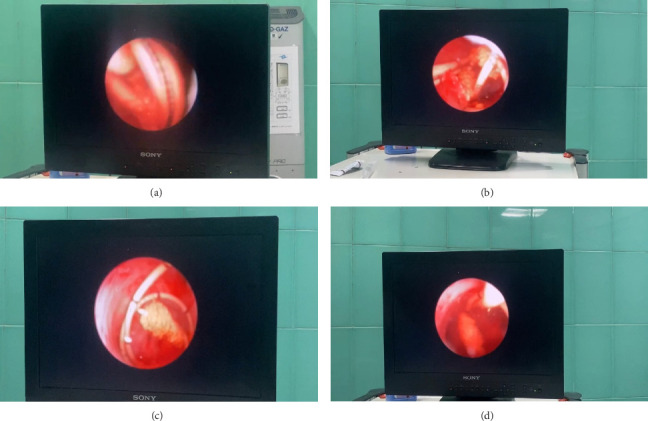
(a–d) Endoscopic view showing the knotted double J stent within the left renal pelvis. The image demonstrates the complex entanglement of the stent, highlighting the challenges encountered during endoscopic intervention for stent removal.

Routine preoperative laboratory tests, including biochemistry, creatinine levels, and urine culture, were normal. Under spinal anesthesia, the patient underwent a cystoscopic procedure using a Storz 22 Fr cystoscope. Initial attempts to remove the left DJ stent with a grasper were unsuccessful. A semirigid Storz 8 Fr ureteroscope was subsequently used under guidance to access the ureter. The procedure revealed significant encrustation along the DJ stent, which was carefully fragmented using a lithoclast from the ureter to the renal pelvis. Lithoclast was preferred over laser fragmentation due to its effectiveness in breaking down the hardened encrustations efficiently, availability in our setting, and cost-effectiveness compared with laser technology. Notably, the upper tip of the stent in the renal pelvis was found knotted (Figure 4).

With precise maneuvers and without cutting the stent, the knot was untangled and the DJ stent was completely removed. A new 5 Fr ureteral DJ stent was placed to ensure drainage. The surgery lasted 60 min. The patient remained under observation for 24 h postoperatively, demonstrating stable creatinine levels and no complications. The patient was discharged in good condition. At the 1-month follow-up, no residual stones or functional abnormalities were detected, and the patient returned to normal activities.

## 3. Discussion

Prolonged indwelling of double-J stents can lead to complications such as urinary tract infections, hematuria, pain, and bladder irritation symptoms [8]. Knotted DJ stents are exceedingly rare, with fewer than 100 cases reported globally [2, 3, 5]. Encrustation adds another layer of complexity, particularly when present alongside knotting [2, 5]. Risk factors for knotting include long stent retention times, improper placement, and anatomical variations such as short ureters [4, 5]. Approximately 90% of the knotted stents occur in the proximal ureter or renal pelvis. While encrustation is often linked to urinary infections, metabolic disorders, or biofilm formation, it is not directly implicated in knotting [2, 5]. Despite significant encrustation observed during ureteroscopy, the preoperative CT scan did not reveal it, likely due to the limited sensitivity of noncontrast CT for radiolucent materials and the stent's orientation. Fluoroscopic and endoscopic evaluations remain crucial for detecting such cases [9–11].

This case demonstrates an effective minimally invasive strategy in the management of knotted and encrusted DJ stents. Unlike conventional strategies where the presence of severe encrustation at the tip of the stent within the renal pelvis often leads to a change in the treatment plan toward more invasive procedures such as PCNL, we opted for a less invasive solution. The use of a semirigid ureteroscope was challenging due to limited maneuverability in the renal pelvis and the knotted stent, but using lithoclast fragmentation, we successfully cleared the encrusted segment, allowing the stent tip to be freed. Following this, the DJ stent on the left side was gently extracted using a grasper without the need for open or percutaneous techniques. This approach highlights the effectiveness of combining advanced endoscopic tools to preserve ureteral integrity and minimize patient morbidity [3–5].

This case aligns with previously reported instances where minimally invasive techniques were successfully employed to manage knotting and encrustation. Semirigid ureteroscopes and lithoclast probes are considered effective tools for stent removal in such cases [3, 5, 7]. Advanced endoscopic techniques often obviate the need for open or percutaneous approaches, reducing patient morbidity and recovery time. However, in cases of extreme encrustation or failed endoscopic attempts, open surgery or percutaneous methods may still be necessary [5].

Studies suggest that a structured stepwise approach, starting with cystoscopic evaluation and progressing to ureteroscopic techniques, is the safest and most effective strategy for managing knotted stents [3, 5, 7]. In this case, the use of a semirigid ureteroscope combined with lithoclast fragmentation allowed successful removal of the stent while preserving ureteral integrity. Fluoroscopic guidance can also aid in visualizing the knot's location and ensuring complete removal, particularly in complex cases but we do not use Fluoroscopic or C arm [3, 5]. This further emphasizes the importance of advanced endoscopic tools in managing such complex cases. This case underscores the importance of timely stent removal to prevent complications and highlights the need for patient education and follow-up systems, such as automated reminders, to minimize the incidence of forgotten stents [4, 7].

## 4. Conclusion

This case highlights the importance of timely DJ stent removal and patient follow-up to prevent complications such as encrustation and knotting. Endoscopic techniques, particularly lithoclast fragmentation combined with semirigid ureteroscopy, offer an effective and minimally invasive solution for managing complex cases.

## Data Availability

The data used to support the findings of this study are available on request from the corresponding author.

## References

[1] Singh I. (2003). Indwelling JJ Ureteral Stents-A Current Perspective and Review of Literature. *Indian Journal of Surgery*.

[2] Jendouzi O., Lamghari A., Jamali M., Harchaoui A., Alami M., Ameur A. (2022). Knotted Double J Ureteral Stent: A Case Report and Literature Review. *Pan African Medical Journal*.

[3] Choo Z. W., Hong S. K., Lee Y. M. (2021). Management of Knotted Ureteral Stent: A Case Report and Comprehensive Review of Literature. *Clinical Case Reports and Reviews*.

[4] Geavlete P., Georgescu D., Mulțescu R., Stanescu F., Cozma C., Geavlete B. (2021). Ureteral Stent Complications: Experience on 50,000 Procedures. *Journal of Medicine and Life*.

[5] Braga M., Frasson M., Vignali A., Zuliani W. (2007). Minimally Invasive Surgery in Urology. *Urology*.

[6] Groeneveld A. (1989). The Role of ESWL in the Treatment of Large Kidney Stones. *Singapore Medical Journal*.

[7] Ray R. P., Mahapatra R. S., Mondal P. P., Pal D. K. (2015). Long-Term Complications of JJ Stent and Its Management: A 5 Years Review. *Urology Annals*.

[8] Geavlete P., Georgescu D., Mulțescu R., Stanescu F., Cozma C., Geavlete B. (2021). Ureteral Stent Complications: Experience on 50,000 Procedures. *Journal of Medicine and Life*.

[9] Tang V. C., Attwell-Heap A. (2011). Computed Tomography Versus Ureteroscopy in Identification of Renal Tract Stone With Ureteral Stent In Situ. *Annals of the Royal College of Surgeons of England*.

[10] Huang J., Wu W., Zhang S. (2021). Characteristics of Double-J Stent Encrustations and Factors Associated With Their Development. *Urology Journal*.

[11] Lam J. S., Gupta M. (2002). Tips and Tricks for the Management of Retained Ureteral Stents. *Journal of Endourology*.

